# Supplementary material for: Structural insights into how DEK nucleosome binding facilitates H3K27 trimethylation in chromatin
Source: Nat Struct Mol Biol. 2025 Feb 21;32(7):1183–92. doi: 10.1038/s41594-025-01493-w (PMC12263440; doi:10.1038/s41594-025-01493-w)
Supplement: Supplementary file 1 — Reporting Summary [file 41594_2025_1493_MOESM1_ESM.pdf]

nature portfolio

Corresponding author(s): Hitoshi Kurumizaka, Yukiko Gotoh

Last updated by author(s): Dec 17, 2024

Reporting Summary

Nature Portfolio wishes to improve the reproducibility of the work that we publish. This form provides structure for consistency and transparency in reporting. For further information on Nature Portfolio policies, see our Editorial Policies and the Editorial Policy Checklist.

Statistics

For all statistical analyses, confirm that the following items are present in the figure legend, table legend, main text, or Methods section.

n/a	Confirmed	
☐	☒	The exact sample size (*n*) for each experimental group/condition, given as a discrete number and unit of measurement
☐	☒	A statement on whether measurements were taken from distinct samples or whether the same sample was measured repeatedly
☐	☒	The statistical test(s) used AND whether they are one- or two-sided<br>*Only common tests should be described solely by name; describe more complex techniques in the Methods section.*
☐	☒	A description of all covariates tested
☐	☒	A description of any assumptions or corrections, such as tests of normality and adjustment for multiple comparisons
☐	☒	A full description of the statistical parameters including central tendency (e.g. means) or other basic estimates (e.g. regression coefficient) AND variation (e.g. standard deviation) or associated estimates of uncertainty (e.g. confidence intervals)
☐	☒	For null hypothesis testing, the test statistic (e.g. *F*, *t*, *r*) with confidence intervals, effect sizes, degrees of freedom and *P* value noted<br>*Give P values as exact values whenever suitable.*
☒	☐	For Bayesian analysis, information on the choice of priors and Markov chain Monte Carlo settings
☒	☐	For hierarchical and complex designs, identification of the appropriate level for tests and full reporting of outcomes
☒	☐	Estimates of effect sizes (e.g. Cohen's *d*, Pearson's *r*), indicating how they were calculated

Our web collection on statistics for biologists contains articles on many of the points above.

Software and code

Policy information about availability of computer code

Data collection	AcquireMP ver. 2023 R1.1, SerialEM ver.3, EPU 3.1, NanoWizard IIR Instrument (JPK)
Data analysis	Relion 3.1.4, Relion 4.0.0, MotionCor2 1.3.2 and 1.4.0, gctf v1.18 and v1.60, ctffind-4.1.14, UCSF ChimeraX 1.2 and 1.6, ISOLDE 1.2 and 1.6, Coot 0.9.3, PHENIX 1.21rc1,UCSF Chimera 1.14, PyMOL, 2.5.5, ImageJ 1.52q, DiscoverMP ver. 2023 R1.2, gwyddion 2.64, RStudio 2022.07.2 +576, FASTX-Toolkit 0.0.14, cutadapt 2.6, bowtie2 2.3.5.1, bedtools2 2.29.0, samtools 1.9, f-seq 1.84, hisat2 2.1.0, featureCounts 1.6.0, ngsplot 2.63, MolProbity in PHENIX 1.21rc1

For manuscripts utilizing custom algorithms or software that are central to the research but not yet described in published literature, software must be made available to editors and reviewers. We strongly encourage code deposition in a community repository (e.g. GitHub). See the Nature Portfolio guidelines for submitting code & software for further information.

Data

Policy information about availability of data

All manuscripts must include a data availability statement. This statement should provide the following information, where applicable:

- Accession codes, unique identifiers, or web links for publicly available datasets
- A description of any restrictions on data availability
- For clinical datasets or third party data, please ensure that the statement adheres to our policy

The cryo-EM reconstructions and atomic models of the DEK2, DEK1 - and H1-nucleosomes have been deposited in the Electron Microscopy Data Bank and the

Protein Data Bank (PDB) under the accession codes: DEK2-nucleosome: EMD-37115 and PDB ID: 8KCY; DEK1-nucleosome: EMD-37121 and PDB ID: 8KD1; H1-nucleosome: EMD-37149 and PDB ID: 8KE0; free-nucleosome (closed): EMD-38443; free-nucleosome (middle): EMD-38444; free-nucleosome (open): EMD-38445. The sequence data have been deposited in the DNA Data Bank of Japan (DDBJ) Sequence Read Archive under the following accession codes: DRA016775 (DEK ChIP-seq using mouse embryonic brain), DRA019123 (DEK ChIP-seq using HEK293 cells), DRA016561 (H3K27me3 ChIP-seq), DRA016776 (H3K27me3 CUT&Tag), DRA016778 (H2AK119ub1 CUT&Tag), DRA016781 (ATAC-seq), and DRA016780 (RNA-seq). We also used published datasets: GSE133391 (H3K27me3 ChIP-seq using HEK293 cells) and GSE249290 (RNA-seq using HEK293 cells). The DEK SAP structure (PDB ID: 2JX3) was downloaded from PDB.

Research involving human participants, their data, or biological material

Policy information about studies with human participants or human data. See also policy information about sex, gender (identity/presentation), and sexual orientation and race, ethnicity and racism.

Reporting on sex and gender	N/A
Reporting on race, ethnicity, or other socially relevant groupings	N/A
Population characteristics	N/A
Recruitment	N/A
Ethics oversight	N/A

Note that full information on the approval of the study protocol must also be provided in the manuscript.

Field-specific reporting

Please select the one below that is the best fit for your research. If you are not sure, read the appropriate sections before making your selection.

☒ Life sciences ☐ Behavioural & social sciences ☐ Ecological, evolutionary & environmental sciences

For a reference copy of the document with all sections, see nature.com/documents/nr-reporting-summary-flat.pdf

Life sciences study design

All studies must disclose on these points even when the disclosure is negative.

Sample size	Sample size calculation of AFM analyses was not conducted, but Particles were collected in numbers comparable to well established experimental approaches.(Wang et al., Biophys. J., 2009). Sample sizes of genome study were chosen as standard in the field (Hirabayashi et al., Nauron, 2009; Eto et al., Nature Communications, 2020). The replicate numbers were based on estimation from previous studies.
Data exclusions	During cryo-EM analyses, bad particles were excluded as shown Extended Data Figures. Other than the cryo-EM analyses, no data was excluded from the analysis.
Replication	The reproducibility of the findings were confirmed by performing at least two independent experiments. All replicated experiments were performed successfully. Cryo-EM analyses were conducted once because the final map already represented the average of a large number of images.
Randomization	Data was not randomized during biochemical analyses. Cryo-EM processing software Relion randomly splits particles into two different half-maps during 3D reconstruction in order to calculate resolution of the 3D volume. Randomization was not relevant to biochemical experiments. For genome study, all sample allocation were randomized.
Blinding	Since there was no subjective allocation in our investigation, blinding was irrelevant to this study

Reporting for specific materials, systems and methods

We require information from authors about some types of materials, experimental systems and methods used in many studies. Here, indicate whether each material, system or method listed is relevant to your study. If you are not sure if a list item applies to your research, read the appropriate section before selecting a response.

Materials & experimental systems

n/a	Involved in the study
☐	☒ Antibodies
☐	☒ Eukaryotic cell lines
☒	☐ Palaeontology and archaeology
☐	☒ Animals and other organisms
☒	☐ Clinical data
☒	☐ Dual use research of concern
☒	☐ Plants

Methods

n/a	Involved in the study
☐	☒ ChIP-seq
☒	☐ Flow cytometry
☒	☐ MRI-based neuroimaging

Antibodies

Antibodies used

anti-H3K27me3 antibody (Cell Signaling #9733) used at a 1:1000 dilution.
anti-H3K27me3 antibody (1E7) used at a 1:1000 dilution.
anti-Rabbit IgG, HRP-Linked F(ab')2 Fragment Donkey (Cytiva #NA9340) used at a 1:3000 dilution.
anti-Mouse IgG HRP Linked F(ab')2 Fragment (Cytiva #NA9310) used at a 1:5000 dilution.
anti-H2B antibody (Cell Signaling #2934) used at a 1:1000 dilution.
anti-rabbit IgG conjugated with Alexa Fluor 488 (Jackson Immuno Research Laboratories, Inc. #111-545-144) used at a 1:500 dilution.
anti-mouse IgG conjugated with Alexa Fluor 647 (Invitrogen, #A32728) used at a 1:5000 dilution.
H2AK119ub1 (Cell Signaling #8240S) used at a 1:100 dilution.
anti-DEK (BD, 610948) used at a 1:500 dilution.
anti-GAPDH (Cell Signaling, 2118S) used at a 1:500 dilution.
anti-H3 (Active Motif, 39763) used at a 1:5000 dilution.
anti–mouse Alexa Fluor 555 (Thermo Fisher Scientific, A-31570) used at a 1:1000 dilution.

Validation

anti-H3K27me3 antibody (Cell Signaling #9733). Western blot of various cell lines was performed. (https://www.cellsignal.com/products/primary-antibodies/tri-methyl-histone-h3-lys27-c36b11-rabbit-mab/9733)
anti-H3K27me3 antibody (1E7). ELISA using the valious H3 N-terminus tail peptides was performed. (https://academic.oup.com/nar/article/39/15/6475/1023574?login=false)
anti-DEK antibody (BD #610948). Western blot of a Jurkat cell lysate was performed. (https://www.bdbiosciences.com/en-br/products/reagents/microscopy-imaging-reagents/immunofluorescence-reagents/purified-mouse-anti-human-dek.610948)
H2AK119ub1 (Cell Signaling #8240S). Western blot of various cell lines was performed. (https://www.cellsignal.com/products/primary-antibodies/ubiquityl-histone-h2a-lys119-d27c4-xp-rabbit-mab/8240)
anti-GAPDH (Cell Signaling, 2118S). Western blot of various cell lines was performed. (https://www.cellsignal.jp/products/primary-antibodies/gapdh-14c10-rabbit-mab/2118)
anti-H3 (Active Motif, 39763). Western blot of a Hela cell lysate was performed. (https://www.activemotif.com/catalog/details/39763)

Eukaryotic cell lines

Policy information about cell lines and Sex and Gender in Research

Cell line source(s)	HEK293: provided by Dr. Tetsu Akiyama, the University of Tokyo
Authentication	The authors declare that the cell lines were authenticated based on their morphology
Mycoplasma contamination	The cell lines were not tested for mycoplasma contamination.
Commonly misidentified lines (See ICLAC register)	HEK293

Animals and other research organisms

Policy information about studies involving animals; ARRIVE guidelines recommended for reporting animal research, and Sex and Gender in Research

Laboratory animals	JCL:ICR (CLEA Japan) and Slc:ICR (Kapan SLC), embryonic day 11 to 12
Wild animals	No wild animals
Reporting on sex	No data analyses based on sex differences. Pooled sample of male and female were used.
Field-collected samples	No field-collected samples
Ethics oversight	Animal Care and Use Committee of The University of Tokyo (approval numbers: P25-8 and P30-4 in the Graduate School of Pharmaceutical Sciences, and 0421 and A2022IQB001 in the Institute for Quantitative Biosciences)

Note that full information on the approval of the study protocol must also be provided in the manuscript.

Plants

Seed stocks	*Report on the source of all seed stocks or other plant material used. If applicable, state the seed stock centre and catalogue number. If plant specimens were collected from the field, describe the collection location, date and sampling procedures.*
Novel plant genotypes	*Describe the methods by which all novel plant genotypes were produced. This includes those generated by transgenic approaches, gene editing, chemical/radiation-based mutagenesis and hybridization. For transgenic lines, describe the transformation method, the number of independent lines analyzed and the generation upon which experiments were performed. For gene-edited lines, describe the editor used, the endogenous sequence targeted for editing, the targeting guide RNA sequence (if applicable) and how the editor was applied.*
Authentication	*Describe any authentication procedures for each seed stock used or novel genotype generated. Describe any experiments used to assess the effect of a mutation and, where applicable, how potential secondary effects (e.g. second site T-DNA insertions, mosiacism, off-target gene editing) were examined.*

ChIP-seq

Data deposition

☒ Confirm that both raw and final processed data have been deposited in a public database such as GEO.

☒ Confirm that you have deposited or provided access to graph files (e.g. BED files) for the called peaks.

Data access links<br>*May remain private before publication.*	Database: the DNA Data Bank of Japan (DDBJ)<br>Raw sequence files: DRA016775 (DEK ChIP-seq), DRA016561 (H3K27me3 ChIP-seq), DRA016776 (H3K27me3 CUT&Tag), DRA016778 (H2AK119ub1 CUT&Tag), DRA016781 (ATAC-seq), and DRA016780 (RNA-seq).<br>Processed files : E-GEAD-680 (DEK ChIP-seq, called peaks, bed files), E-GEAD-681 (DEK ChIP-seq, bigwig files), E-GEAD-681 (H3K27me3 CUT&Tag, called peaks, bed files), E-GEAD-682 (H2Aub CUT&Tag, called peaks, bed files), E-GEAD-683 (ATAC-seq, called peaks, bed files), E-GEAD-678 (H3K27me3 ChIP-seq, called peaks, bed files), E-GEAD-679 (H3K27me3 ChIP-seq, bigwig files), DRA019123 (DEK ChIP-seq using HEK293 cells), E-GEAD-874 ((DEK ChIP-seq using HEK293 cells, bigwig files)
Files in database submission	DEK ChIP-seq, rep 1<br>DEK ChIP-seq, rep 3<br>H3K27me3 CUT&Tag_Con, rep 1<br>H3K27me3 CUT&Tag_Con, rep 2<br>H2AK119ub1 CUT&Tag_Con, rep 1<br>H2AK119ub1 CUT&Tag_Con, rep 2<br>ATAC-seq_Con, rep 1<br>ATAC-seq_Con, rep 2<br>ATAC-seq_Con, rep 3<br>ATAC-seq_Con, rep 4<br>H3K27me3 CUT&Tag_Con, rep 1<br>H3K27me3 CUT&Tag_Con, rep 2<br>H3K27me3 CUT&Tag_Con, rep 3<br>DEK ChIP-seq using HEK293, rep 1<br>DEK ChIP-seq using HEK293, rep 2
Genome browser session<br>(e.g. UCSC)	No longer available.

Methodology

Replicates	Two (DEK ChIP-seq, H3K27me3 CUT&Tag, H2Aub CUT&Tag), three (H3K27me3 ChIP-seq), four (ATAC-seq)
Sequencing depth	151 bp paired end (DEK ChIP-seq, H3K27me3 CUT&Tag, H2Aub CUT&Tag), 100 bp paired end (ATAC-seq), 50 bp single end(H3K27me3 ChIP-seq)
Antibodies	DEK (BD, 610948)<br>H3K27me3 (CST, 9733S)<br>H2Aub (CST, 8240S)
Peak calling parameters	Software: f-seq<br>Parameters: t5 (DEK ChIP-seq), t7 (ATAC-seq), t9 (H3K27me3, H2Aub CUT&Tag)
Data quality	Significant signals in positive control regions (Hoxa cluster for H3K27me3 and H2Aub, Actb for ATAC-seq) and lower signals in negative control regions (Actb for H3K27me3 and H2Aub and Hoxa cluster for ATAC-seq)
Software	FASTX-Toolkit 0.0.14, cutadapt 2.6, bowtie2 2.3.5.1, bedtools2 2.29.0, samtools 1.9, f-seq 1.84, hisat2 2.1.0, featureCounts 1.6.0, ngsplot 2.63
