# Supplementary material for: Structural insights into how DEK nucleosome binding facilitates H3K27 trimethylation in chromatin
Source: Nat Struct Mol Biol. 2025 Feb 21;32(7):1183–92. doi: 10.1038/s41594-025-01493-w (PMC12263440; doi:10.1038/s41594-025-01493-w)
Supplement: Supplementary file 4 — Unprocessed images for Fig. 6b. [file 41594_2025_1493_MOESM4_ESM.pdf]

Supplementary_Data_1

Free poly-nucleosome | DEK + poly-nucleosome | H1 + poly-nucleosome

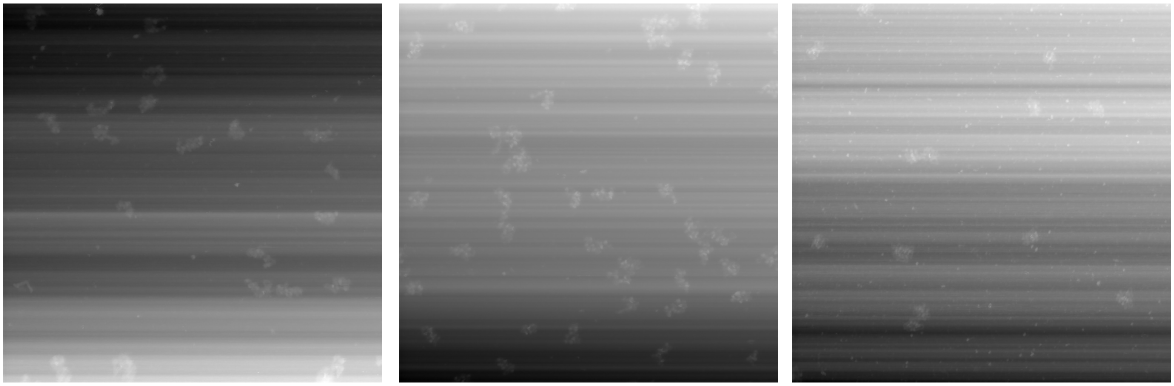

Unprocessed images for Figure 6b
