# Supplementary figures and images for: Structural insights into how DEK nucleosome binding facilitates H3K27 trimethylation in chromatin
Source: Nat Struct Mol Biol. 2025 Feb 21;32(7):1183–92. doi: 10.1038/s41594-025-01493-w (PMC12263440; doi:10.1038/s41594-025-01493-w)

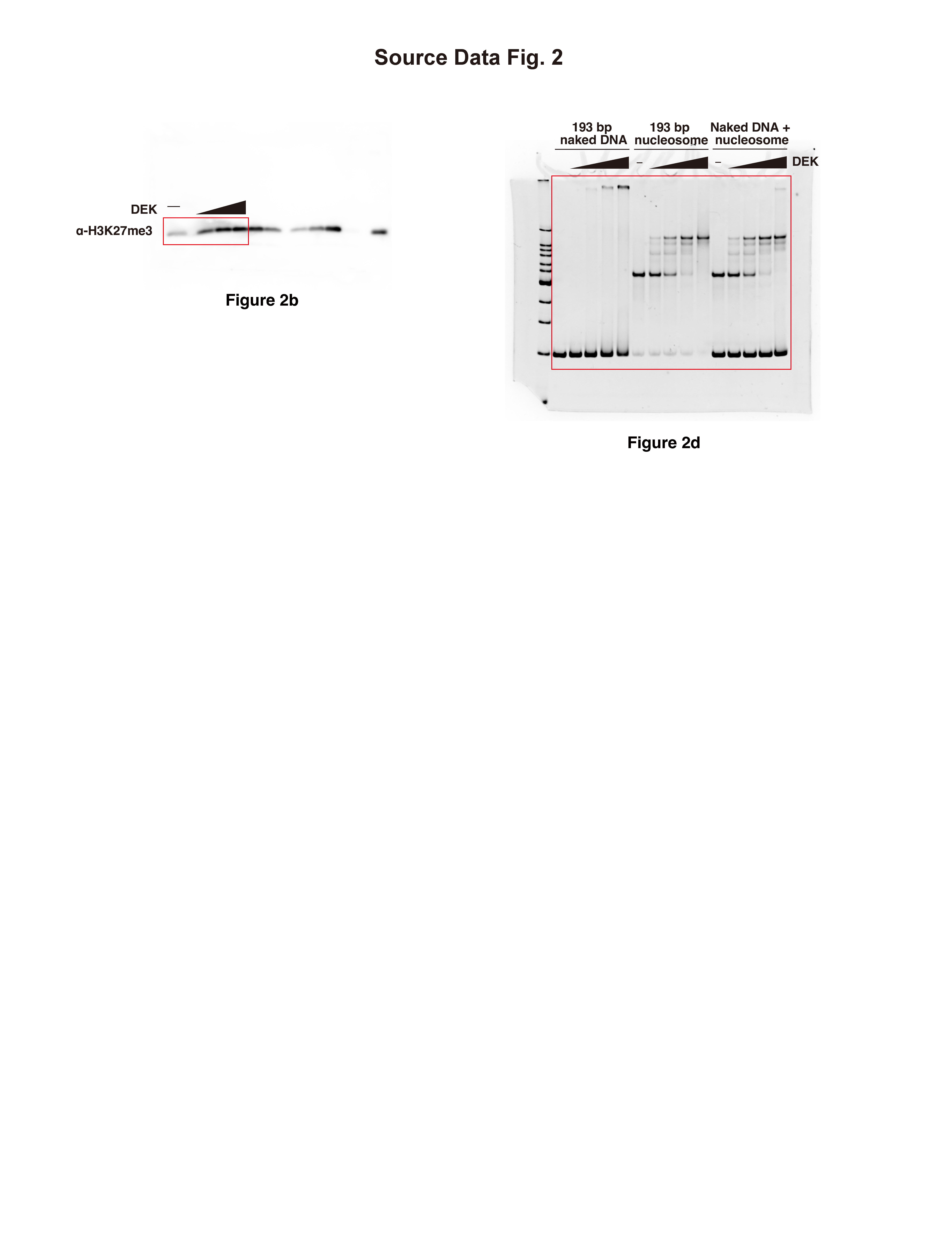

Supplement: Supplementary file 5 — Unprocessed western gels. [file 41594_2025_1493_MOESM5_ESM.jpg]

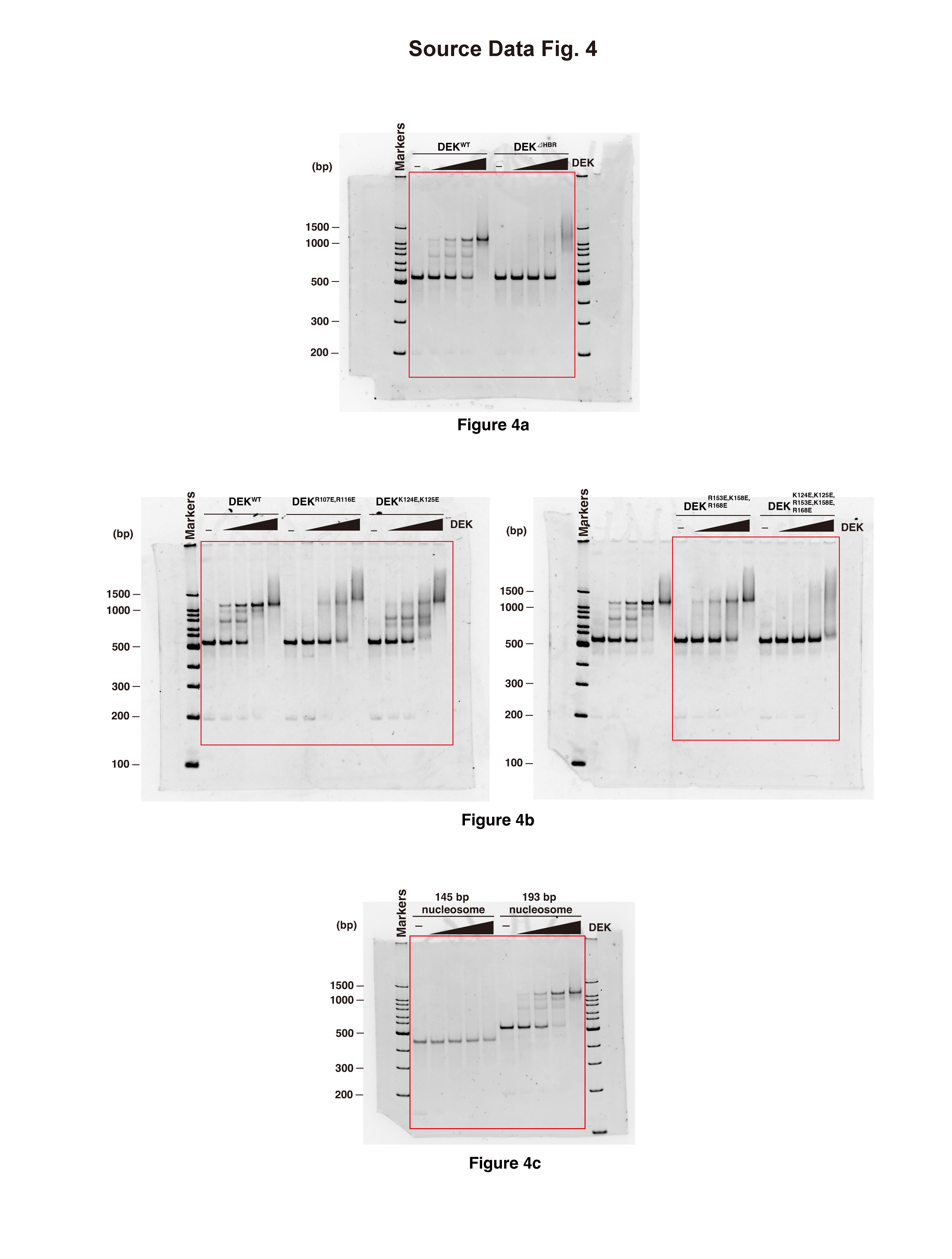

Supplement: Supplementary file 7 — Unprocessed western gels. [file 41594_2025_1493_MOESM7_ESM.jpg]

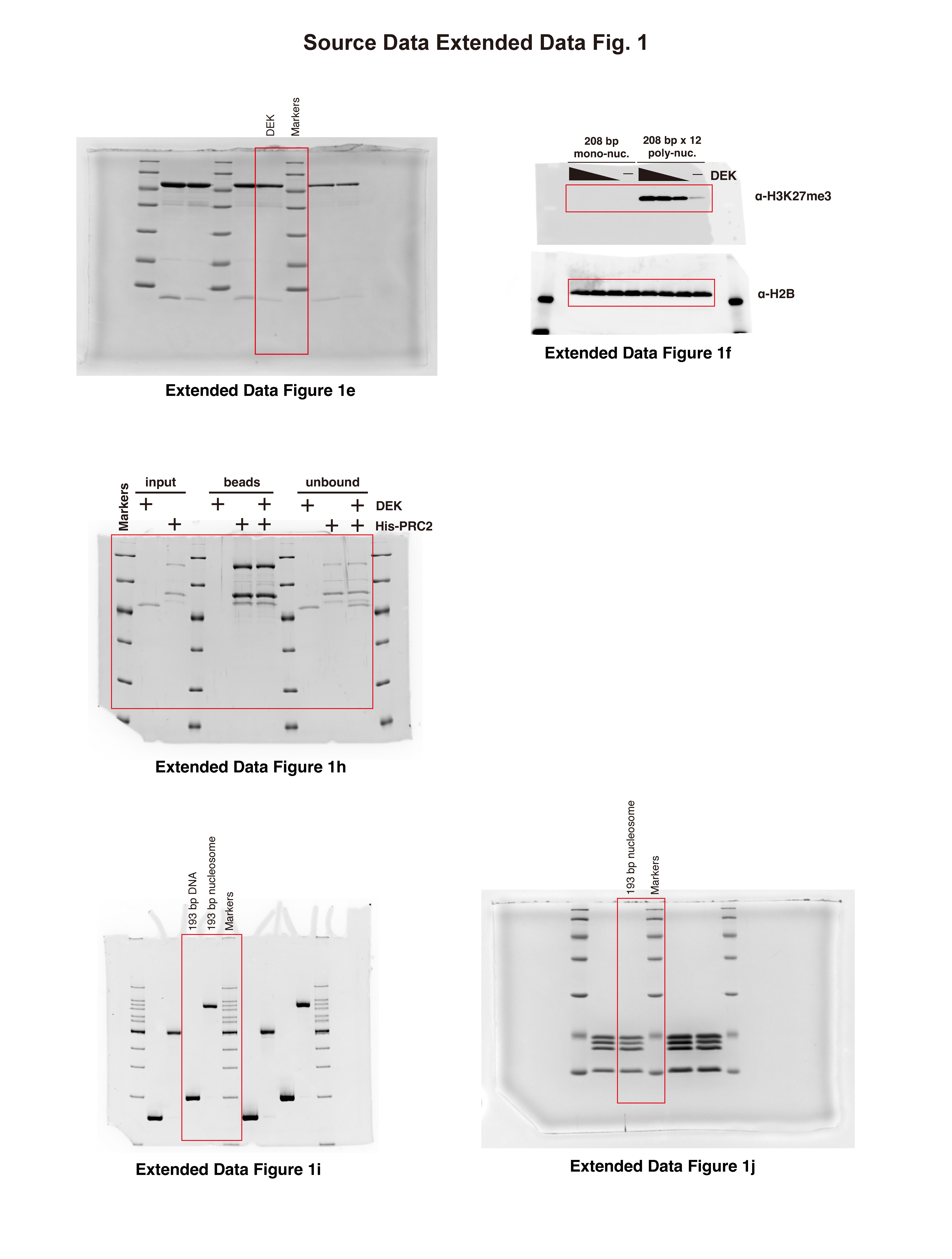

Supplement: Supplementary file 9 — Unprocessed western blots and gels. [file 41594_2025_1493_MOESM9_ESM.jpg]

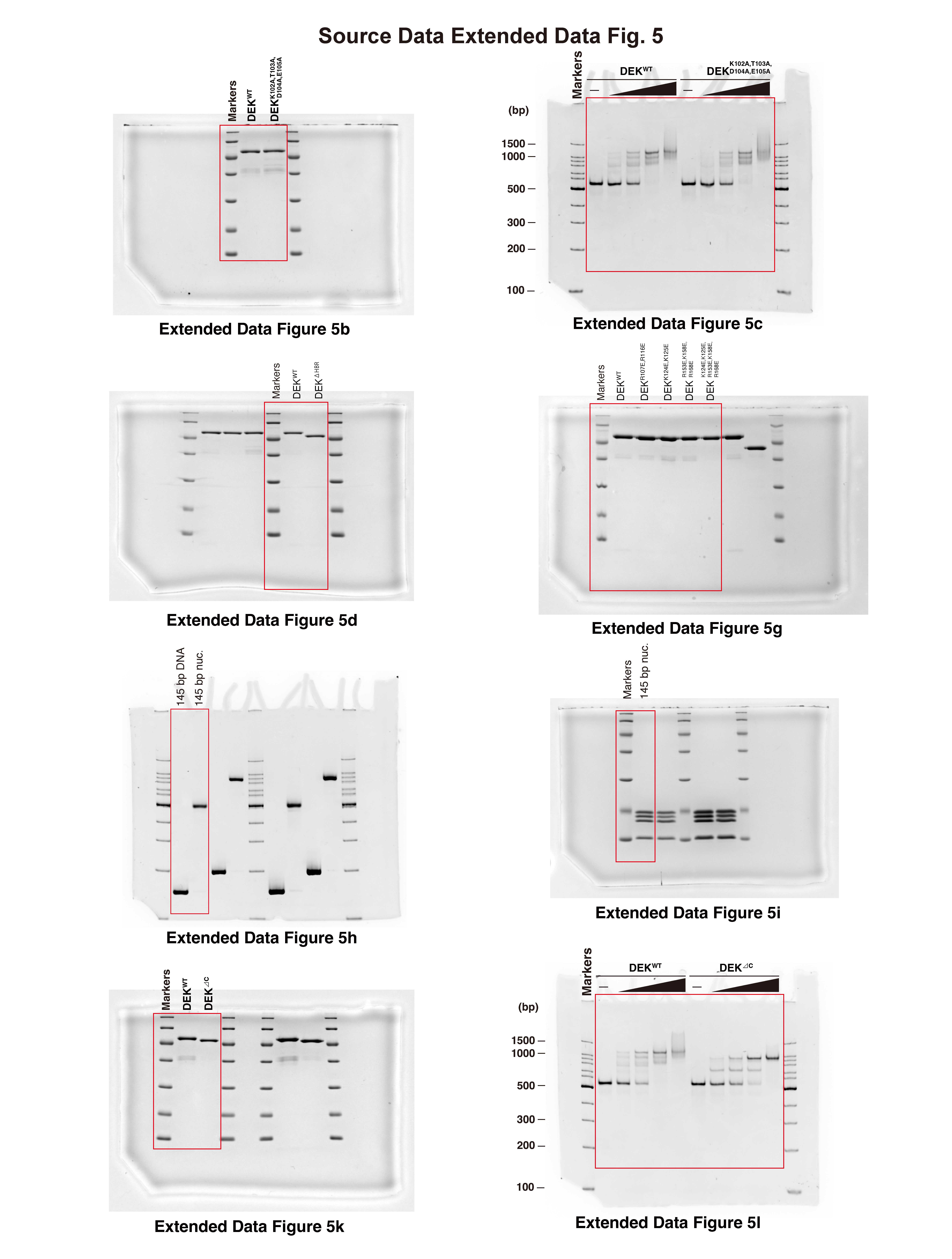

Supplement: Supplementary file 10 — Unprocessed western gels. [file 41594_2025_1493_MOESM10_ESM.jpg]

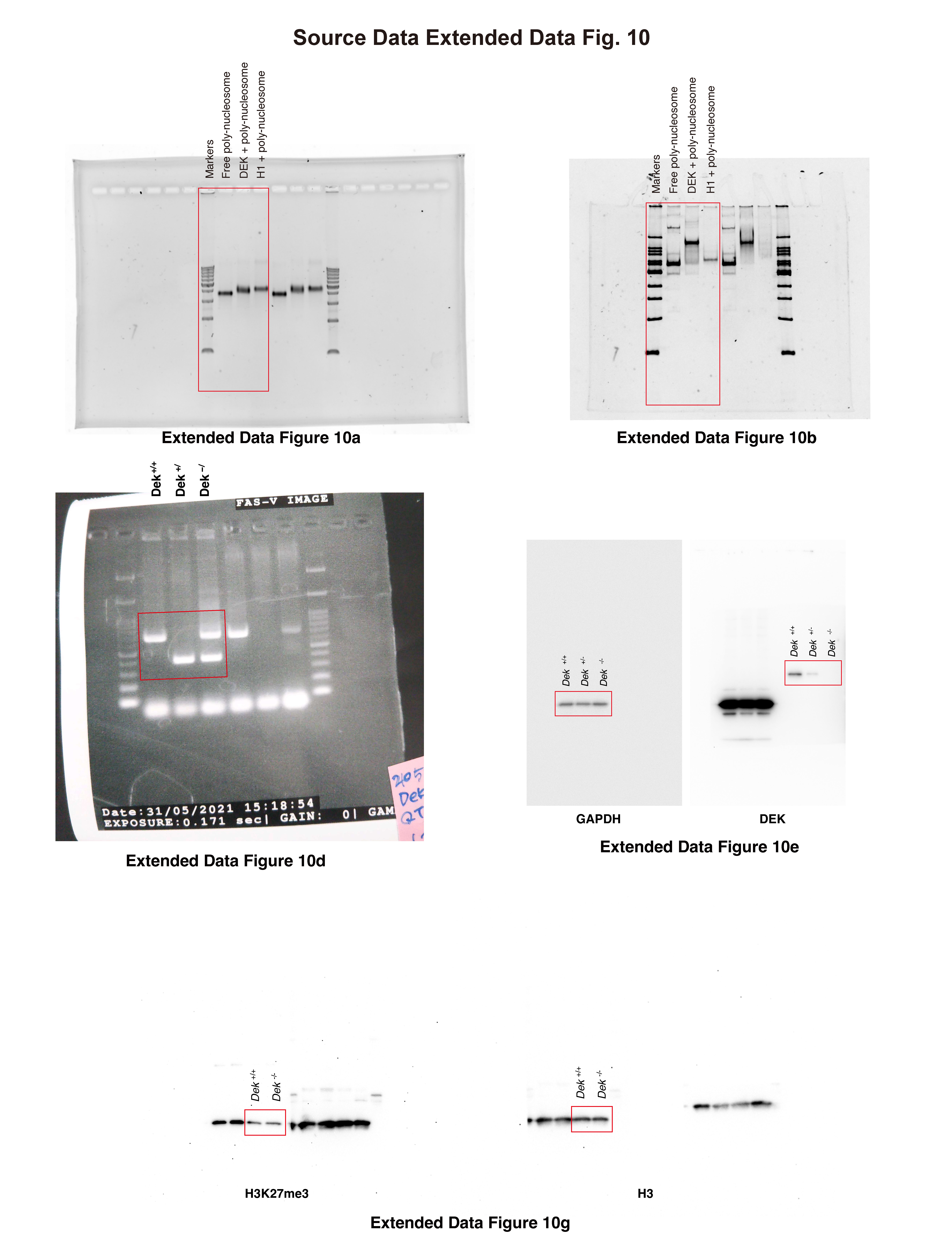

Supplement: Supplementary file 11 — Unprocessed western blots and/or gels. [file 41594_2025_1493_MOESM11_ESM.jpg]
